# Development of higher-order thinking skills in nursing students through online problem-based assessment

**DOI:** 10.4102/hsag.v28i0.2423

**Published:** 2023-10-24

**Authors:** Olivia B. Baloyi

**Affiliations:** 1 School of Nursing and Public Health, College of Health Sciences, University of KwaZulu-Natal, Durban, South Africa

**Keywords:** higher order thinking skills, psychiatry, nursing students, online assessment, problem based assessment

## Abstract

**Background:**

The development of higher-order thinking skills (HOTS) in problem-based learning (PBL) is not confined to teaching and learning but extends to authentic assessment methods, similar to real-life situations. The assessments aligned to PBL attempt to eliminate the students’ tendency towards memorisation. Rather, it instils and encourages their ability to analyse, interpret, synthesise, and evaluate knowledge and its sources.

**Aim:**

The study had two primary aims: (1) to describe undergraduate nursing students’ experiences of an online problem-based assessment (PBA), and (2) to explore how online PBA assessment contributed to the development of undergraduate student nurses’ HOTS.

**Setting:**

An urban-based South African higher education institution (HEI) in KwaZulu-Natal, Durban, South Africa.

**Methods:**

A descriptive, exploratory qualitative approach was used. The target population was 4th-year psychiatric nursing students (*N* = 39) studying for the degree of Bachelor of Nursing at the preselected university, utilising two focus groups (*n* = 5, *n* = 7). Data were analysed through content analysis using the clinical reasoning model as a framework.

**Results:**

Three categories (trigger problem, re-visioning the cues, treatment direction) and seven sub-categories (trigger problem posing, early cue identification, cue interpretation and clustering, focussed cue investigation, information processing and interpretation, reprioritise hypotheses, and diverse intervention[s]) emerged.

**Conclusion:**

Psychiatry, Nursing and Midwifery practices require a practitioner skilled in HOTS to provide quality, efficient and cost-effective patient care.

**Contribution:**

The findings in this study can benefit nursing education, particularly learning interruptions in HEIs.

## Introduction

The wide use of problem-based learning (PBL) began at McMaster University in the mid-1960s. It aimed to imbue in students problem-solving skills for operationalisation in the ‘real world’, namely the clinical environment (Schmidt 2012). Problem-based learning is defined as a student-centred pedagogical approach triggered by an authentic, ill-structured problem that requires the collaborative, active engagement of the students, who are understood as self-directed, active knowledge seekers and creators of knowledge when arriving at an academic resolution (Dharma, Tasrikah & Churiyah 2020). The teaching and learning process involved in PBL requires students to learn to be internally motivated, and self-driven in their pursuit of education and collaborate with others to become ‘life-long learners’ (Yew & Goh 2016). The real-life and contextual nature of PBL develops higher-order thinking skills (HOTS) and contributes towards making learning meaningful (Frey 2018). The HOTS are not simply problem-solving skills but include applying knowledge and involving reflective thinking to arrive at well-reasoned practice decisions (Frey 2018).

The development of HOTS in PBL is not confined to teaching and learning but extends to authentic assessment methods, similar in nature to real-life situations (Frey 2018; Whitlock & Nanavati 2013). The assessments aligned with PBL attempt to eliminate the students’ tendency towards memorisation, but instead instil and encourage their ability to analyse, interpret, synthesise, and evaluate knowledge and its sources (Baloyi & Mtshali 2018a,b). The focus of PBL is the problem-solving process involved in acquiring knowledge to facilitate the arrival of the outcome, thereby contributing to lifelong learners as opposed to the focus of traditional education on content-driven assessment. Problem-based learning assessment methods aimed at developing HOTS include reflective journals, portfolios, group presentations, and the triple jump exercise (Mtshali & Middleton 2010). As an example, the triple jump exercise aligns with PBL, consisting of three steps: (1) Problem definition; (2) information search and study; (3) problem synthesis formulation and intervention (Chian, Bridges & Lo 2019).

Many assessment methods, such as the triple jump exercise, have relied on face-to-face interactions between students and academics. However, the restrictions accompanying the coronavirus disease 2019 (COVID-19) pandemic caused significant disruptions in global education by suspension of in-person classes by higher education institutions (HEIs), affecting how teaching, learning and assessments were conducted (United Nations 2020). In response to the COVID-19 pandemic, the United Nations (2020) proposed strategies to:

[*R*]eimagine education and accelerate a change in learning and teaching by seizing the opportunity to find new ways to address the crisis and to bring about a set of solutions previously considered difficult or impossible to implement. (United Nations 2020:4)

Chian et al. (2019) highlight how the online learning and teaching environment differs from the face-to-face context; therefore, tailored assessment strategies for the online learning environment should be offered.

Problem-based learning guides the discipline under discussion. During the pandemic, the nursing discipline faced tailor-making assessments that allowed for the development of HOTS but recognised the restrictions imposed by the COVID-19 regulations and face-to-face interactions between academics and students. Before the pandemic, nursing faculty used the triple jump exercise as an assessment method to develop HOTS. Consequently, academics involved in reconfiguring student assessment to the online format and teaching the undergraduate psychiatric nursing science module designed an alternate, innovative online approach to the triple jump exercise.

## Aim

The study had two primary aims: (1) to describe undergraduate nursing students’ experiences of an online problem-based assessment (PBA); and (2) to explore how online PBA contributed to the development of undergraduate student nurses’ HOTS.

## Research methods and design

Weaver and Olson (2006) define a paradigm as a ‘set of suppositions and practices that structure inquiry within a direction by providing lenses, frames and operations through which exploration is executed’. A social constructivist paradigm was used for this study. Scholars in support of this paradigm believe in the deep understanding of a concept and they explore the understanding of the world in which they live and work (Rahi et al. 2019). Aligned with this paradigm, a qualitative approach that was descriptive and exploratory attempted to answer the aforementioned two primary research objectives. Qualitative research is used to discover in-depth information (Biggam 2011). For this reason, the authors considered this approach appropriate in this study, allowing them to engage in discussions with participants to uncover in-depth knowledge regarding participation in an online problem-based assessment and how it contributed to their developing HOTS. Furthermore, the authors aimed to use probing and open-ended questions to describe and gain a deeper understanding of the phenomenon under study, hence the use of an exploratory and descriptive research design (Weller et al. 2018).

### Study setting

The study setting was an urban-based university in KwaZulu-Natal, Durban, South Africa. After successfully completing the university baccalaureate nursing programme, graduates are eligible for registration with the South African Nursing Council as a nurse (General, Community and Psychiatry) and midwife (South African Nursing Council [SANC] 2005). Over three decades ago, the nursing faculty at the said university realised the inadequacy of a content-driven curriculum in preparing competent, responsive and relevant graduates (Mthembu, Mtshali & Frantz 2014). Subsequently, the faculty at the university shifted from a content-driven curriculum to a context-driven curriculum, which was ultimately guided by the principles of a problem-based, competency-oriented, student-centred curriculum aimed at producing competent, responsive and relevant nurses and midwives (Mtshali & Gwele 2016). The ultimate goal of teaching and learning in the study setting is to instil discipline-specific and transferable competencies in students.

### Population, sampling procedure and participants’ description

In a research study, the inclusion and exclusion criteria constitute the eligibility criteria used to qualify or disqualify the target population (Patino & Ferreira 2018). In this study, the target population was 4th-year students (N = 39) studying full-time for the degree of Bachelor of Nursing at a large public university in South Africa. Completing degree requirements allows registration as a Nurse (General, Community and Psychiatry) and Midwife. Potential participants had to be registered for the Psychiatric Nursing Science module, had or were participating in online PBA, could speak English, and were willing to provide written informed consent. Exclusion criteria included students who had not had or were not registered for the Psychiatric Nursing Science module, were registered but did not participate in the online PBA, or did not consent to be included in the study.

*Purposive sampling, a non-probability sampling method*, was used (Allen 2017). Such a sampling method allowed for selecting participants with in-depth knowledge of the issues under discussion. This sampling approach was most likely to contribute to the richness of data while simultaneously addressing the aim, objectives and study questions (Allen 2017). In congruence with the *Protection of Personal Information Act* (POPIA), section 12 (De Waal 2022), all 4th-year students registered for the Psychiatric Nursing Science module in the Bachelor of Nursing degree were verbally informed about the study by one of the researchers during a class contact.

*Recruitment:* The 4th-year Bachelor of Nursing students enrolled in the psychiatric nursing module were informed verbally by O.B.B. about the study. Although O.B.B. was a faculty member at the study site, she was not involved in teaching psychiatric nursing students, making it easier for the students to relate to her. All participants were each given information letters before voluntarily signing consent to participate in the study.

### Data collection and analysis process

Data collection occurred through two focus group discussions (FGDs) (*n* = 5, *n* = 7), referred to as FGD 1 and FGD 2 in the results section and document analysis (DA) of online assessments from the learning management system (LMS) site, referred to as DA in the results section. Document analysis of online problem-based assessment was undertaken concurrently with FGDs. All participants (*N* = 12) were informed that data collected in the study would be coded and kept confidential, using pseudonyms during the Zoom web conferencing sessions. The rationale for using FGD interviews was to obtain rich detail by ensuring that core research concepts were covered and encouraging participants to relate their perspectives in a conversational manner (Nyumba et al. 2018). The following open-ended question(s), as indicated in the interview guide, guided the discussions during the FGD: ‘Describe your experience of the online problem-based assessment’. Probing questions were used to ensure that the students provided a stepwise narrative on how the problem-based assessment helped them to develop HOTS, including:

- What did you experience as challenging in the online problem-based assessments?
- What did you experience as mentally stimulating (enjoy academically) in the online problem-based assessments?
- In which part of the problem-based assessment did you find yourself engaging with HOTS (e.g. problem-solving, critical thinking)?

To ensure credibility (Muijeen, Kongvattananon & Somprasert 2020), both researchers (O.B.B. & M.A.J.) were present in the two FGDs. Both researchers are academics at the study site. However, only M.A.J. was actively involved in teaching and assessing the study participants. Because O.B.B. did not have any teaching or assessment contact with the study participants, she assumed the lead facilitator role, with MAJ as co-facilitator. This arrangement prevented the possibility of coercion (Råheim et al. 2016) between M.A.J., as the subject teacher, and the study participants. Both researchers O.B.B. and M.A.J. were female and had experience in qualitative data collection as researchers themselves and research supervisors.

#### Focus group discussions

Focus group data were collected over 1 week during the 5th week of the semester. Both FGDs were kept to the recommended duration of between 1 and 2 h, which enhanced rich discussions (Muijeen et al. 2020).

#### Document analysis

Online DA occurred through the LMS site, where the students completed their assessments. O.B.B. and M.A.J. participated in DA of the online assessments from the LMS site to identify any evidence of the student’s development of HOTS as they engaged with the PBA. Such evidence included, but was not limited to, the student’s ability to identify the problems from the presenting situation, understand the links between ideas, approach problems consistently and systematically, analyse and discover facts through concept connection, and skills such as structured questioning, problem-solving, idea generation, analytical thinking, creative thinking, among others.

#### Online problem-based assessment

The newly designed online PBA involved a paper based case with Parts A–D accessible through three online assessment links on the LMS site. Students had to type a response to the different parts of the PBA on the LMS site. Time allowances accommodated the students moving in and out of the respective online assessment links on the LMS site. The students were provided with 20 min (inclusive of five minutes of reading time) to complete Part A, 40 min for Part B and 90 min for Parts C and D.

Part A involved posing a problem to the students through an ill-structured presenting problem with limited information about a mental healthcare user. The students had to engage their cognitive skills and generate multiple hypotheses through early cue identification, clustering and interrogation of the complex authentic presenting problem (Baloyi & Mtshali 2018b). The students were assessed on the relevance, comprehensive nature and broad-based approach of the problem or issue identified. Upon closure of the online assessment part A link, and following a 5min break, all students moved to a new online assessment link for Part B.

Part B centred on focused cue investigations (Baloyi & Mtshali 2018c). It required the student to cluster the cues from their initial hypothesis created in Part A and accordingly type the critical inquiry-oriented questions for which they needed answers to refine their hypothesis (Baloyi & Mtshali 2018a,c). In addition, they were required to explain the knowledge, which guided their questioning. As a final step in Part B, students were asked to provide a well-formulated revised hypothesis. Assessment for these two sections of Part B centred on the accuracy, appropriateness, and holistic approach of the questions, and the ability to state the knowledge that guided the questions. The inquiry process was measured through the systematic or unsystematic way in which the students stated their questions. Their ability to reflect critically and self-correct utilising new information was measured through the revised hypothesis. After a short comfort break of 10 min, the students returned through a new online assessment link to address Parts C and D.

In Part C, the undergraduate students were presented with the structured scenario and its relevant information, completing any gaps left in the ill-structured problem. The students were expected to read the scenario and, through information discrimination and interpretation, distinguish between relevant and irrelevant data (Baloyi & Mtshali 2018c).

Thereafter, in Part D, in line with the importance of reflection and self-evaluation in the development of HOTS (Baloyi & Mtshali 2018c), based on the provided scenario, the students were asked to identify the key gaps they had failed to enquire about through their generated questions in Part B. Assessment centred on their ability to evaluate their earlier formulations critically.

Furthermore, in Part D, in the development of clinical reasoning skills, the students were required to provide a nursing diagnosis of the patient in the provided online scenario and reflect on it compared with their revised hypotheses generated in Part B. Thereafter, the student selected relevant information and identified at least one, and a maximum of five, mental health problems from the scenario. Assessment of Parts C and D lay in assessing the student’s ability to successfully complete four tasks, namely: (1) provide a well-formulated online nursing diagnosis reflective of concepts, theories and nursing models that underpinned the intervention; (2) rank problems in order of priority with rationale as well as the inter-relatedness of problems; (3) describe nursing interventions linked to the diagnosis; and (4) resolve more than one issue.

#### Data analysis

Data were analysed from the two focus groups (*N* = 12) and the online assessments from the LMS site (*N* = 12) by O.B.B. and M.A.J. using the content analysis approach outlined by Elo and Knygas (2008), framed within the clinical reasoning model developed by (Baloyi & Mtshali 2018c). The data collector (O.B.B.) listened carefully to the audio recording soon after each FGD to gain a general sense of the whole. This was followed by O.B.B. carefully transcribing the data from both FGDs. O.B.B. read through the transcripts repeatedly, word for word, to gain a full understanding of their meaning. Similar sentences and words were identified and coded. Following this, the related codes were grouped together and allocated into subcategories. Then, the analysis proceeded to the labelling of subcategories, which were then sorted into categories. The programme NVivo version 12 qualitative data analysis software was used to further organise and support the analysis to ensure credibility. O.B.B. and M.A.J. met several times to discuss and interpret finalised categories and subcategories and reach a consensus prior to meeting with the participants to confirm preliminary findings. A similar process was followed with the DA of online problem-based assessment. Both FGDs and online assessments from the LMS site were analysed for evidence of the student’s development of HOTS through their ability to apply the clinical reasoning core concepts as indicated in the clinical reasoning model developed by (Baloyi & Mtshali 2018a,b).

### Trustworthiness

Trustworthiness was established using Lincoln and Guba’s (1985) strategies of credibility, dependability, confirmability and transferability. Member checking was used to ensure credibility, and researchers validated collected data against emerging results with the participants and matched it against verbatim transcripts (López-Zerón, Bilbao-Nieva & Clements 2021). On several occasions, the researchers, O.B.B. and M.A.J. met to discuss and agree on the emerging sub-categories and categories to reduce their own bias (Mackieson, Shlonsky & Connolly 2019). In order to adhere to the principles of confirmability, an audit trail was generated (Anney 2014). Firstly, field notes were kept along with memos to authenticate transcribed data. Secondly, FGDs were recorded and transcribed verbatim. Thirdly, data were collected until data saturation was achieved, and lastly, data coding was performed separately by OBB and MAJ to reach solidarity and ensure inter-coder agreement on the gathered findings (Carminati 2018). Dependability was ensured when the study’s findings were consistent and reliable (Forero et al. 2018). Qualitative research experts carried out data quality audits, and checks to ensure dependability. In addition, the researcher provided a thick and dense description of the research methodology and data. The qualitative research approach deals with small samples. Therefore, generalisability poses a challenge (Carminati 2018). Therefore, in order to ensure transferability to other contexts, a thick description of the research procedures, study setting, context and findings is provided. This would serve as a guide and enable other researchers and scholars to evaluate the study’s applicability to their own context (Carminati 2018).

### Ethical considerations

Gatekeeper permission was obtained from the study university’s Registrar, followed by submitting the proposal to the Human Social Science Research Ethics Committee of the selected university for revision and approval. The study commenced once ethical clearance (HSSREC/00004304/2022) was granted. Because of data being collected after hours and over mealtime, the students were given a voucher for food to compensate for the inconvenience. The participants were informed that their participation was entirely voluntary and they could withdraw from the study without any consequences (Resnik 2015). Furthermore, participants were assured that their information would be protected through coding to maintain anonymity, and the data and/or information would be stored under lock and key in the principal investigator’s (O.B.B.) office.

All FGDs were group sessions conducted through Zoom web conferencing because of the COVID-19 pandemic; therefore, participants were urged to switch their cameras off to maintain anonymity during the discussions and pseudonyms were used. The participants were fully informed that there was no monetary compensation. However, the findings in this study will benefit stakeholders and policymakers towards establishing effective online teaching during pandemics or any other situation, which hinders face-face teaching. Participants were made aware that physical risks were not foreseen, but minimal emotional and psychosocial harm could be experienced as a result of revealing academic information they might consider private. A student counsellor employed by the university was recruited to the study to ensure that students who become emotional during the FGDs were voluntarily offered support and counselling. Therefore, services offered were at no financial cost to the students. The students were provided with the counsellor’s contact number. All participants connected from campus using university’s free Wi-Fi. There were no financial implications to connecting on Zoom.

## Results

The response rate was 30.7% (N = 12), involving 2 male and 10 female undergraduate final-year nursing students. Ages ranged from 21 to 42 years, with a mean age of 23.67 years. Participants provided information through two focus discussion groups (FGD1 *n* = 5; FGD2 *n* = 7) (Table 1).

**TABLE 1:** Demographic profile of participants (N = 12).

FGD	Date and duration	Participant number	Sex	Age (years)	Full-or part-time status	Race
FGD1	23 June 2022, 45 min	P1	F	22	Full-time	W
		P2	F	23	Full-time	A
		P3	M	21	Full-time	A
		P4	F	22	Full-time	A
		P5	F	23	Full-time	I
FGD2	29 June 2022, 60 min	P1	F	22	Full-time	A
		P2	F	23	Full-time	A
		P3	M	42	Full-time	W
		P4	F	21	Full-time	I
		P5	F	21	Full-time	I
		P6	F	22	Full-time	A
		P7	F	22	Full-time	A

FGD, Focus Group Discussion; F, Female; M, Male; W, White people; A, African people; I, Indian people.

### Category 1: Trigger problem

The first category showed the students’ alignment with the clinical reasoning model designed by Baloyi and Mtshali (2018c) with evidence of three sub-categories (trigger problem posing, early cue identification, and cue interpretation).

#### Sub-category 1.1: Trigger problem posing

The first sub-category demonstrated the student’s ability to generate knowledge from a broad, ill-defined problematic scenario. At this stage, the students were able to express their need to develop structured questions generated from the ill-defined, ill-structured problem and identified the need to develop the hypotheses (Table 2).

**TABLE 2:** Categories and sub-categories following data analysis of Focus Group Discussion (*N* = 12).

Categories	Sub-categories
Category 1: Trigger problem	Sub-category 1.1: Trigger problem posing
	Sub-category 1.2: Early cue identification
	Sub-category 1.3: Cue interpretation and clustering
Category 2: Re-visioning the cues	Sub-category 2.1: Focussed cue investigation
	Sub-category 2.2: Information processing and interpretation
Category 3: Treatment direction	Sub-category 3.1: Reprioritise hypotheses
	Sub-category 3.2: Diverse interventions

Note: Student discussion: critical enquiry 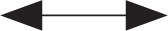 self-correction 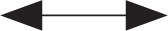 correct diagnosis

The next expressions by students were consistent with the findings in the online PBA:

‘We were provided with a scenario, but it did not have all the information, which was tricky for me because I had to come up with a hypothesis.’ (FGD1, P1, F)

‘That’s what we always get for PBA we are not given enough content in the scenario at the beginning…that’s how it supposed to be it has always been like that our lecturers have always said it is meant for us to ask relevant questions and come up with hypothesis.’ (FGD1, P3, M)

Similarly, online assessments from the LMS site by the researchers, as per DA, showed the following:

‘Impaired coping strategy related to not focusing at his work manifested by receiving a warning.’ (DA, P4, F, 21)

‘Mr. Sydney is displaying a frightened facial expression or affect which can mean he having visual hallucination (seeing something that is scary).’ (DA, P5, F, 21)

#### Sub-category 1.2: Early cue identification

Students struggled with early cue identification. According to them, they were unable to dissect the ill-defined presenting situation and could not highlight the key issues, which negatively impacted their ability to identify early cues. The following verbal extracts provide evidence that students struggle with early cue identification:

‘Without fully understanding what is happening with the case, early cue identification was not possible, it was difficult I really struggled, I just guessed.’ (FGD 1, P3, M)

‘I could not interpret the case, I was unable to highlight the key issues emerging from the scenario hence I could not come up with early cues.’ (FGD2, P7, F)

‘During our classes the process of early cue identification is simple because our lecturers ask us some trigger questions which I find helpful as they assist us to think outside the box, but with the problem-based assessment I’m expected to engage with the questioning myself which made this phase of early cue identification very hard.’ (FGD2, P6, F)

#### Sub-category 1.3: Cue interpretation and clustering

For this sub-category, the students were expected to group the cues, which seemed to be related to generating multiple hypotheses. In addition, at this stage, the students should demonstrate their ability to utilise numerous reasoning processes, such as intuition, narrative and analytical thinking (Tanner 2006). However, this was not evident in this study, as indicated in the following excerpts:

‘I knew what was expected of me at this stage, but it was not happening, I just could not think, it was hard for me group together all the concepts which were talking to each in order to make sense of the case.’ (FGD2, P5, F)

‘Our facilitator emphasised to us that the only way to formulate a hypothesis, is if we carefully interpret what is happening in the case they gave us, group together issues which are related and then a hypothesis will come out, but I could not think like this during the assessment.’ (FGD2, P5, F)

‘The phases of this problem-based assessment are linked together, if you fail in one the entire assessment become flawed, I did not know the key presenting issues from the case, which is why cue clustering and interpretation was challenging for me, I omitted this stage.’ (FGD2, P1, F)

### Category 2: Re-visioning the cues

The students were expected to think critically in terms of re-visioning the cues. However, it is here that they developed anxiety about the assessment, which suppressed their ability to ask the relevant questions, leading to the realisation that they were failing to meet the assessment requirements (Table 2). The verbal extracts demonstrated that despite prior preparation through dry run by psychiatric lecturers for the upcoming PBA, they had been hindered by the constraints posed by their anxiety:

‘It was very difficult for me to think critically as to what is happening in this scenario, I could not ask questions I started becoming anxious.’ (FGD2, P4, F)

‘Yes, I formulated the hypotheses which was not very hard I but now I could not ask relevant questions I became anxious because I was like stuck.’ (FGD1, P4, F)

‘We had a dry-run with our lecturers with a different scenario off course but during the actual assessment I just had a mind block not knowing what questions to ask, yes I attempted to ask questions but I knew they were not relevant questions, anxiety about the entire assessment was mounting, the questioning part became even more harder.’ (FGD2, P7, F)

‘I was just anxious at this stage, I don’t know why I just could not ask structured questions, my questioning was not relevant at all I knew it.’ (FGD1, P3, M)

Students further verbalised that the level of anxiety they experienced impeded the ability to use technology, exacerbated by the clinical manifestations of anxiety such as pounding heartbeat and sweating hands. Time also seemed to be moving faster than usual:

‘With the high anxiety I had, I found it hard to move my mouse.’ (FGD2, P5, F)

‘My hands were shaking and sweating, they were slippery - I could not grasp the mouse …’ (FGD1, P5, F)

‘… Oh, my word, when I looked at my watch I was only left with 2 minutes for Part B of the PBA to close and I was not done, my hands could not move, I froze and looked at my laptop screen.’ (FGD2, P5, F)

‘… I also could not finish, Part B just closed as I was busy, I lost track of time.’ (FGD2, P3, M)

Poor critical thinking about the case was evident in the student’s inability to ask relevant questions. The following extract confirms the irrelevant questions asked by the students:

‘Family history: [*H*]as any family member had mental illness? Did any family member pass away in a traumatic manner?’ (DA, P2, F, 23)

#### Sub-category 2.1: Focussed cue investigation

Five students reflected that their level of anxiety appeared to interfere with their ability to focus on the questions. This further interfered with their ability to understand the problem in totality. The outcome was that the questions asked were in a haphazard manner, and rationale was omitted or incorrect reasoning was provided. The ability to arrive at a hypothesis assisted in reaching a diagnosis. However, when a student could not reach a diagnosis, questioning was not systematic and did not make sense. The following extracts indicate how the students struggled in this section of the online assessment process:

‘I was not focussed [*on*] how I was asking the questions.’ (FGD2, P1, F)

‘I was all over the place…maybe is because I was too anxious, I don’t know… when I thought I asked a good question, I could not think of the reason why I asked that question …’ (FGD2, P6, F)

‘For me I could not come up with the correct diagnosis even if I got it right at the end, it was just from guessing because I could not risk not writing anything I didn’t want to lose marks.’ (FGD2, P3, M)

‘Me too I guessed the diagnosis my questions were not focussed, I didn’t even know why I was asking them…during the dry run I remember Dr XX told that if we ask relevant questions with rationale it will be easy to arrive at the diagnosis.’ (FGD1, P5, F)

‘… I just guessed the diagnosis, I had no choice - I had to guess, the level of the questions I asked was not sufficient enough to arrive at the correct diagnosis …’ (FGD1, P3, M)

The inability to ask focused questions because of anxiety was not valid for all students. Some students coped with this section of the online PBA, as shown in the following quote:

‘I think I managed to ask relevant questions, even though I was not sure that all my rationale [*were*] correct, I was able to come up with the correct diagnosis.’ (FGD2, P4, F)

The following excerpt demonstrates the omission of a rationale; this student was asking questions to gain an understanding of the case:

‘Due to lack of communication, MHCU [*mental health care user*] might lose their relationships as evidenced with MHCU having difficulties with girlfriend.’ (DA, P7, F, 22)

The majority of students alluded to the mounting levels of anxiety they experienced when unable to meet the assessment requirements. While four of the students agreed with their colleagues regarding anxiety and assessment requirements, they added that their level of anxiety was superimposed by the lack of congruency between teaching and learning and assessment methods at the study site. The following verbal extracts support their assertion:

‘Yes, I was anxious about the assessment but I think if I was better prepared I could have kept my calm, normally during classroom presentations we do power point presentation, with not so good engagement … like in the classroom I’m not pushed to do what I’m supposed to be doing in the assessment’ assessment.’ (FGD1, P4, F)

‘Like my colleague said, in the classes we present, we are not offered an opportunity to engage with an ill-defined case no hypothesis formulation no asking of critical questions like we do in our assessments this gave me a lot of anxiety in this exam.’ (FGD1, P3, M)

‘We just have to be taught the way we are going to be assessed … it will be very helpful for us because at least we would have practiced more this way of learning and exam anxiety would have been lesser or maybe not even there.’ (FGD2, P1, F)

Some students appreciated that their rationale was irrelevant as involved in the following statement:

‘Is the MHCU coping financially and how does he spend his money? [*C*]ould the MHCU be relapsing? Is the MHCU on any substances such as drugs and alcohol? Does the MHCU have a good appetite and is the MHCU sleeping well?’ (DA, P3, M)

#### Sub-category 2.2: Information processing and interpretation

Most of the participants battled with this section of information processing and interpretation and expressed difficulty in processing the cues they had collected. As a result of their their inability to apply HOTS, they failed to fully understand the problem. They could not differentiate relevant from irrelevant information, impeding their reflective ability. Reflection is perceived as important during this phase because it allows the students to identify gaps in their thinking process (Baloyi & Mtshali 2018c). Similarly, the students could not reflect and thus they failed to self-correct. The following extracts attest to this:

‘It was very hard for me [*to*] process the information. I could not see what is relevant and what is irrelevant in this case, because I failed to collect sufficient information in the other parts of the assessment.’ (FGD 1, P3, M)

‘… I could not even identify the gaps in my own knowledge, I didn’t know what I know and what I didn’t know, I tried to reflect on this but could not identify any knowledge gaps, they were there but I didn’t know them if I may put it that way.’ (FGD1, P5, F)

### Category 3: Treatment direction

Students registered for the Psychiatric Nursing Science module who had moved through to the final stage of the PBA did not believe they had reached the stage of treatment direction. This is authenticated in the third category and resulted in two sub-categories.

#### Sub-category 3.1: Reprioritise hypotheses

The students knew they were expected to reprioritise the hypothesis they had formulated but found it difficult. The following statement extracts show how students struggled in this area:

‘What is good about the PBA is that it offers us with an opportunity to go back and relook at our initially formulated hypotheses and reorganize them, but this was difficult for me in this assessment I could not reprioritized my hypothesis, I just guessed and that was it.’ (FGD 1, P3, M)

‘… mainly because each step leads into the next, so when one is poorly answered it affect[*ed*] the rest … I did not have enough answers from the asked question to revise my initially formulated hypothesis.’ (FG2, P2, F)

In the online extracts, all but one student showed difficulties in reprioritising the hypothesis and appeared unable to address the early signs of relapse despite the increase in medication. Furthermore, many of the students focussed on the sexual dysfunction of chlorpromazine.

#### Sub-category 3.2: Diverse interventions

Students were aware of the required thinking process but identified partial problems instead of seeing problems in totality. For example, students wanted to treat insomnia separately rather than seeing its role in contributing to early relapse. This is what the students said:

‘I could not manage the patient as a whole, because I did not know the diagnosis, instead just to get some marks, I managed some of the problems the patient presented with like insomnia.’ (FGD2, P1, F)

‘… I knew that at this stage of the assessment I’m supposed to provide interventions as per patient diagnosis but because I failed to critically think throughout the assessment, I did not know the diagnosis, and could not see the patient problem in totality.’ (FGD 1, P3, M)

‘… I opted to manag[*ed*] the signs and symptoms rather than the diagnosis.’ (FGD1, P4, F)

## Discussion

The study allowed for the exploration and description of undergraduate psychiatric nursing students’ experiences of an online mental health PBA and its contribution to developing HOTS. The principles of a problem-based, competency-oriented and student-centred curriculum are utilised at the HEI in this study (Mtshali & Gwele 2016), coupled with authentic assessment to develop students’ HOTS.

Participants were aware of the PBA’s teaching, learning and educational intention. They appreciated the ‘puzzling’ ill-defined case and viewed it as authentic. Despite the purposeful omission of vital information, participants recognised that the scenario was intentionally ill-defined and intended as a trigger to interrogate, dissect and highlight key issues. Such activity is essential in developing HOTS (Baloyi & Mtshali 2018c). Part A of the PBA required the students to generate multiple tentative hypotheses (Dolmans et al. 2005; Mtshali & Middleton 2010). Most students believed they had successfully achieved this requirement, and written responses on the LMS site confirmed that the students could formulate hypotheses.

However, as the PBA progressed, the evidence of students’ success with the PBA dwindled. After Part A, students knew they had to move a step further by asking questions to improve their understanding of the problem. Interestingly, they knew that these were not just questions but focused systematic questions with justifications. At this stage, most of the student participants realised that they could not remain focused on questioning and rationalising the questions. The inability to use their various reasoning processes, such as intuition, and narrative and analytical thinking (Tanner 2006) was evident at this stage, and it negatively impacted their continued focus on cues provided in the PBA. Unlike the requirements of Mtshali and Middleton (2010), they could not think logically, their questioning was not focused and lacked appropriate evidence-based knowledge. This appeared to be a result of not identifying early cues nor interpreting and clustering the cues. There was also the problem of time constraints. Several students noted that they ran out of time and could not complete the task, which was a source of stress. When moving to the next activity, the time constraint in the previous session could have provoked a stressful response preventing students from providing the best possible answers. Additionally, the students failed to self-correct, refine the initial hypotheses or develop multi-dimensional, theoretically sound nursing responses and anticipated health outcomes (Baloyi & Mtshali 2018c; Mtshali & Middleton 2010).

High anxiety and nervousness resulted from participants’ inability to self-correct and envision the clues and/or prompts provided. This negatively impacted their ability to engage with, and use, the identified clues.

Engagement with the clues provided would have guided logical thinking and the ability to explore the presenting problem in more depth by asking focused clinical questions. This particular study finding is supported by Baloyi and Mtshali (2018c), who both agree that during the focused cue investigations, students should reflect and ask direct questions about the clues provided to better understand the presented case and facilitate gathering additional data.

Participants also reported more intrusive thoughts and impaired cognitive performance resulting in decreased assessment performance when anxiety levels were high (Azimi 2016). Those with higher anxiety observed an increased pounding heartbeat, sweating hands, and a ticking clock, consistent with the literature (Azimi 2016).

In agreement with Pinnock et al. (2019), it was evident at this stage of cue-revisioning coupled with anxiety that the students were unable to process information using their reasoning and critical thinking skills, referred to in this study as information processing and interpretation. The students could neither interpret the collected data nor discriminate relevant from irrelevant data. Their inability to distinguish relevant from irrelevant data affected their ability to reflect on the cues collected as per Pinnock and colleagues (2019). It is through reflection that the students could identify knowledge gaps in their thinking processes (Baloyi & Mtshali 2018c).

However, in this study, reflection through ‘reflecting thinking’, which is a moment of silence allowing learners to engage with invisible cognitive processes and a necessary ingredient for the development of HOTS, was not evident (Baloyi & Mtshali 2018c; Delany & Golding 2014).

Because of their inability to reflect and reconsider the cues collected, the students could not define the gaps in their critical thinking and reasoning processes (Yazdani, Hosseinzadeh & Hosseini 2017). Thus, they were unable to reprioritise the hypotheses and consequently could not accept or refute the hypothesis. Furthermore, the students failed to understand the case better (Mc Tiernan, Smith & Walsh 2007), resulting in a missed opportunity for individual information searching. According to (Schmidt & Mamede 2015), information searching and self-study is the critical step in the PBA. Students can consult the literature to correctly diagnose the patient. Because the students could not identify their individual learning issues and failed to reformulate a case using the newly acquired information (Mc Tiernan et al. 2007), they were unable to implement a comprehensive mental healthcare plan.

Anxiety affected not only the students’ thinking and reasoning processes but also their use of technology.

Students must practise managing their anxiety before the assessment (Azimi 2016). The student’s anxiety levels influenced their ability to use their computers. Simple activities were challenging, and time started to move on, adding to their anxiety and interfering with their thinking abilities. Some participants experienced what they referred to as ‘brain freeze’. They could not arrive at the correct mental health diagnosis for the patient. The assessment lost relevance as a ‘journey to problem resolution’ (Mc Tiernan et al. 2007). Some students resorted to guessing to obtain marks and pass the assessment, and some acknowledged that if they passed, ‘*it was a pass’* without any understanding.

Furthermore, the students could not gather and evaluate information or generate ideas and assumptions from multiple perspectives to produce a well-reasoned revised analysis with understanding. Instead, they delivered fragmented care instead of a comprehensive approach. The outcome was despite the students being afforded an opportunity for an assessment dry-run, which allowed for practice under the academician’s supervision.

Interestingly, the students were concerned about the disjuncture between teaching, facilitation of learning, and assessment methods in the study setting. The students preferred a critical inquiry method of teaching and learning, as it is an expectation during the assessment process. Unity in this process, according to the students, would have allayed their level of anxiety, which was brought about by not knowing how to respond. It is preferable throughout the 4 years of an undergraduate programme that case-based teaching be used, with the opportunity to interrogate the case study. Teaching and assessment need to be correlated to allow for the preparation of the assessment (Shepard 2019).

## Conclusion

The development of HOTS through an online problem-based assessment is not confined to psychiatry. It can be applied to the advancement of general nursing and midwifery undergraduate and postgraduate learners. Developing a community of scholars with HOTS is a much-needed ability in a post-modernism society with its changed learning landscape. Psychiatry, Nursing, and Midwifery practices require a practitioner skilled in HOTS to provide quality, efficient and cost-effective comprehensive patient care.

### Limitations

Generalisability of the study findings is limited because, firstly, the study was only conducted in one institution. Secondly, the study only focused on the 4th-year undergraduate nursing students registered for the Psychiatric Nursing Science module, making it difficult to generalise the finding to all levels of students. Thirdly, the reliability and validity of the online PBA assessment were not verified.

### Recommendation

The development of HOTS through online problem-based assessments is understudied, especially within nursing practice. Therefore, the authors of this article recommend further research in this regard. Furthermore, the study suggests that nursing education policymakers and curriculum designers encourage and support online assessments beyond the pandemic. The study recommends further research to test the developed online problem-based assessment’s usability, validity and reliability.
